# Supplementary figures and images for: The fungal expel of 5-fluorocytosine derived fluoropyrimidines mitigates its antifungal activity and generates a cytotoxic environment
Source: PLoS Pathog. 2022 Dec 27;18(12):e1011066. doi: 10.1371/journal.ppat.1011066 (PMC9829169; doi:10.1371/journal.ppat.1011066)

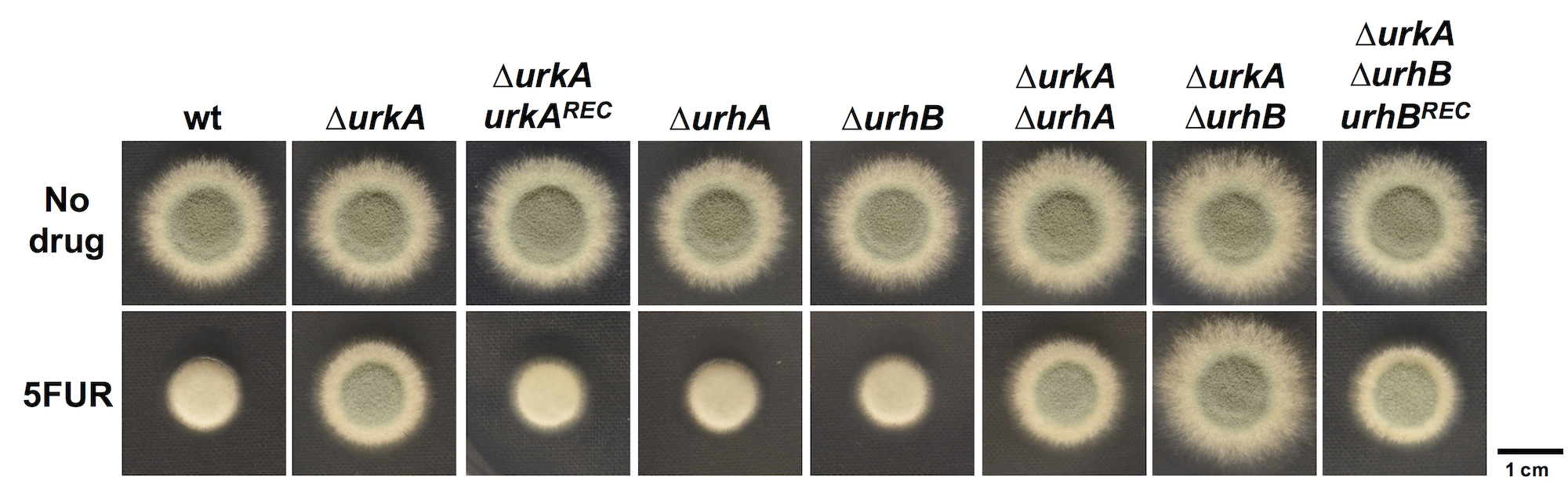

Supplement: S1 Fig — 5FUR resistance of deletion mutants as well as complemented versions (REC) was assessed on solid medium supplemented with 500 μg/mL of 5FUR. (TIF) [file ppat.1011066.s001.tif]

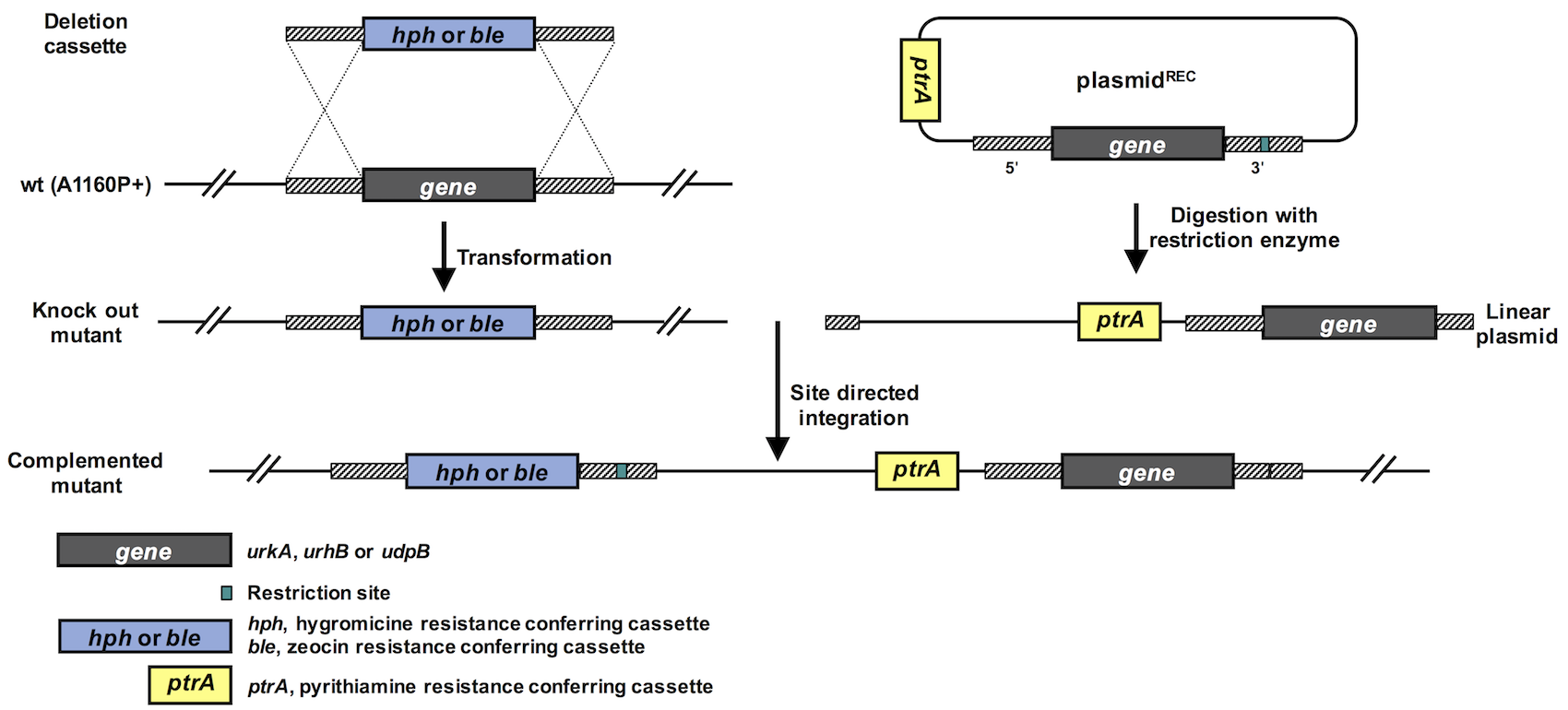

Supplement: S2 Fig — Plasmid for the reconstitution were linearized for site-directed insertion at the corresponding deletion locus. (TIF) [file ppat.1011066.s002.tif]
