# Supplementary material for: The fungal expel of 5-fluorocytosine derived fluoropyrimidines mitigates its antifungal activity and generates a cytotoxic environment
Source: PLoS Pathog. 2022 Dec 27;18(12):e1011066. doi: 10.1371/journal.ppat.1011066 (PMC9829169; doi:10.1371/journal.ppat.1011066)
Supplement: S1 Table — (A) BLASTP analysis suggested one Urk1p ortholog in A. fumigatus. (B) Two orthologs of S. cerevisiae Urh1p were predicted, which we termed UrhA and UrhB. Despite the higher homology, resistance analysis (Figs 1 and S1) confirmed uridine nucleosidase activity for UrhB but not UrhA. (DOCX) [file ppat.1011066.s003.docx]

**S1 Table. Protein-based BLAST analyses (**[**https://blast.ncbi.nlm.nih.gov/Blast.cgi**](https://blast.ncbi.nlm.nih.gov/Blast.cgi)**) of *S. cerevisiae* (S288C) uridine kinase Urk1p (YNR012W) and uridine nucleosidase Urh1p (YDR400W) against *A. fumigatus* (A1163). (A)** BLASTP analysis suggested one Urk1p ortholog in *A. fumigatus*. **(B)** Two orthologs of *S. cerevisiae* Urh1p were predicted, which we termed UrhA and UrhB. Despite the higher homology, resistance analysis (**Figs 1** and **S1**) confirmed uridine nucleosidase activity for UrhB but not UrhA.

**A**

| **Description** | **Score** | **E-value** | **Length (aa)** | **QC** | **% Identity** | **Gene ID** | **Termed** |
| --- | --- | --- | --- | --- | --- | --- | --- |
| Uridine kinase, putative | 338 | 4e-112 | 453 | 90% | 41.81% | AFUB_022460 | UrkA |

**B**

| **Description** | **Score** | **E-value** | **Length (aa)** | **QC** | **% Identity** | **Gene ID** | **Termed** |
| --- | --- | --- | --- | --- | --- | --- | --- |
| Uridine nucleosidase Urh1, putative | 308 | 5e-104 | 358 | 93% | 33.52% | AFUB_005870 | UrhA |
| Nucleoside hydrolase, putative | 169 | 2e-48 | 522 | 88% | 17% | AFUB_011230 | UrhB |
