# Supplementary material for: The fungal expel of 5-fluorocytosine derived fluoropyrimidines mitigates its antifungal activity and generates a cytotoxic environment
Source: PLoS Pathog. 2022 Dec 27;18(12):e1011066. doi: 10.1371/journal.ppat.1011066 (PMC9829169; doi:10.1371/journal.ppat.1011066)
Supplement: S2 Table — Proteins with a putative purine nucleoside phosphorylase and uridine phosphorylase domains (PNP_UDP_1; PF01048) are highlighted in bold. (DOCX) [file ppat.1011066.s004.docx]

**S2 Table. Domain analyses performed with the InterPro database (**[**https://www.ebi.ac.uk/interpro/**](https://www.ebi.ac.uk/interpro/)**) revealed 10 proteins carrying a predicted nucleoside phosphorylase domain (IPR000845) in *A. fumigatus* A1163.** Proteins with a putative purine nucleoside phosphorylase and uridine phosphorylase domains (PNP_UDP_1; PF01048) are highlighted in bold.

| **Gene ID** | **Accession Uniprot** | **Description Interpro** |
| --- | --- | --- |
| AFUB_017990^a^ | B0XT47 | TPR_REGION domain-containing protein |
| AFUB_033090^a^ | B0XVN7 | Pfs and NB-ARC domain protein |
| **AFUB_047570** | **B0XX34** | **PNP_UDP_1 domain-containing protein** |
| AFUB_074680^a^ | B0Y7R0 | S-methyl-5'-thioadenosine phosphorylase |
| AFUB_079240^a^ | B0Y910 | ANK_REP_REGION domain-containing protein |
| AFUB_091840 | B0YB77 | ANK_REP_REGION domain-containing protein |
| AFUB_092960^a^ | B0YCR8 | Purine nucleoside phosphorylase |
| AFUB_097420 | B0YE07 | Pfs, NACHT and Ankyrin domain protein |
| AFUB_097850^a^ | B0YE50 | ANK_REP_REGION domain-containing protein |
| **AFUB_097990**^a^ | **B0YE64** | **PNP_UDP_1 domain-containing protein** |

^a^hits obtained by DELTA-BLAST.
