# Supplementary material for: The fungal expel of 5-fluorocytosine derived fluoropyrimidines mitigates its antifungal activity and generates a cytotoxic environment
Source: PLoS Pathog. 2022 Dec 27;18(12):e1011066. doi: 10.1371/journal.ppat.1011066 (PMC9829169; doi:10.1371/journal.ppat.1011066)
Supplement: S3 Table — Bliss scores were generated to assess potential synergism between CLG and fluoropyrimidines. For drug combinations yielding the 10 highest scores, MICs were visually determined and used for the calculation of the Fractional Inhibitory Concentration Index (FICI). Checkerboard assays were performed in RPMI and evaluated after 24 h (A) and 48 h (B). (DOCX) [file ppat.1011066.s005.docx]

**S3 Table. CLG exerts synergistic interaction with each 5FC, 5FU and 5FUR.** Bliss scores were generated to assess potential synergism between CLG and fluoropyrimidines. For drug combinations yielding the 10 highest scores, MICs were visually determined and used for the calculation of the Fractional Inhibitory Concentration Index (FICI). Checkerboard assays were performed in RPMI and evaluated after 24 h **(A)** and 48 h **(B)**.

**A**

|  |  |  |  | **24 h** | | |  |  |  |  |
| --- | --- | --- | --- | --- | --- | --- | --- | --- | --- | --- |
| **5FC** | **CLG** | **Score** | **5FU** | | **CLG** | **Score** | | **5FUR^c^** | **CLG** | **Score** |
| 50 | 25 | **72.25^a,b^** | 12.5 | | 25 | **79.76^a,b^** | | 1000 | 12.5 | **52.95^a^** |
| 50 | 6.25 | **59.68** | 6.25 | | 25 | **75.98^a,b^** | | 1000 | 25 | **49.68^a^** |
| 50 | 12.5 | **59.63^a,b^** | 12.5 | | 12.5 | **71.52^a,b^** | | 1000 | 6.25 | **47.42** |
| 25 | 25 | **54.13^a,b^** | 25 | | 3.13 | **65.96^a^** | | 2000 | 6.25 | **46.78^a^** |
| 100 | 6.25 | **44.66^a^** | 25 | | 25 | **65.10^a^** | | 2000 | 12.5 | **46.29^a^** |
| 50 | 3.13 | **43.79** | 25 | | 0.78 | **61.80^a^** | | 500 | 25 | **42.17^a^** |
| 100 | 25 | **41.16^a^** | 12.5 | | 3.13 | **60.97** | | 250 | 25 | **41.10^a^** |
| 100 | 3.13 | **39.57^a^** | 25 | | 12.5 | **59.37^a^** | | 2000 | 3.13 | **40.47** |
| 100 | 12.5 | **37.91^a^** | 25 | | 1.56 | **58.53^a^** | | 31.25 | 25 | **39.99** |
| 100 | 1.56 | **32.85** | 12.5 | | 6.25 | **56.13^a,b^** | | 62.5 | 25 | **39.75^a^** |

**B**

|  |  |  |  | **48 h** | | |  |  | | |  |
| --- | --- | --- | --- | --- | --- | --- | --- | --- | --- | --- | --- |
| **5FC** | **CLG** | **Score** | **5FU** | | **CLG** | **Score** | | | **5FUR^c^** | **CLG** | **Score** |
| 50 | 50 | **81.77^a^** | 12.5 | | 25 | **87.02^a,b^** | | | 500 | 50 | **62.73^a^** |
| 100 | 25 | **77.49^a,b^** | 12.5 | | 50 | **84.85^a^** | | | 250 | 50 | **58.37^a^** |
| 100 | 50 | **77.23^a^** | 25 | | 25 | **68.98^a,b^** | | | 31.25 | 50 | **52.85** |
| 50 | 25 | **76.18^a,b^** | 25 | | 50 | **65.59^a^** | | | 2000 | 50 | **50.46^a^** |
| 25 | 50 | **73.53^a^** | 25 | | 12.5 | **57.84^a,b^** | | | 125 | 50 | **43.26** |
| 100 | 12.5 | **61.34** | 6.25 | | 50 | **57.03^a^** | | | 62.5 | 50 | **43.18** |
| 200 | 25 | **56.41^a^** | 12.5 | | 12.5 | **48.88** | | | 1000 | 50 | **41.82^a^** |
| 200 | 50 | **55.77^a^** | 50 | | 25 | **46.10^a^** | | | 2000 | 25 | **41.17^a^** |
| 200 | 12.5 | **52.58^a^** | 50 | | 50 | **43.85^a^** | | | 31.25 | 100 | **19.45^a^** |
| 200 | 6.25 | **48.94^a^** | 6.25 | | 25 | **42.54** | | | 500 | 100 | **18.21^a^** |

^a^drug combinations were visual MICs were determined.

^b^drug combinations were a synergistic FICI value (≤0.5) was obtained.

^c^no FICI was determined for 5FUR as the drug itself does not lead to a full growth inhibition.
