# Supplementary material for: The fungal expel of 5-fluorocytosine derived fluoropyrimidines mitigates its antifungal activity and generates a cytotoxic environment
Source: PLoS Pathog. 2022 Dec 27;18(12):e1011066. doi: 10.1371/journal.ppat.1011066 (PMC9829169; doi:10.1371/journal.ppat.1011066)
Supplement: S4 Table — (DOCX) [file ppat.1011066.s006.docx]

**S4 Table. Strains used in this study.**

| **Strain** | **Genotype** | **Reference** |
| --- | --- | --- |
| A1160P+ (wt) | *∆ku80::pyrG* | (1) |
| *∆fcyA* | *∆fcyA::hph* | (2) |
| *∆uprt* | *∆uprt::ble* | (2) |
| *∆urkA* | *∆urkA::hph* | This study |
| *∆urhA* | *∆urhA::hph* | This study |
| *∆urhB* | *∆urhB::hph* | This study |
| *∆urkA∆urhA* | *∆urkA::hph∆urhA::ble* | This study |
| *∆urkA∆urhB* | *∆urkA::hph∆urhB::ble* | This study |
| *∆urkA urkA^REC^* | *∆urkA::hph urkA^REC^*, *ptrA* | This study |
| *∆urkA∆urhB urhB^REC^* | *∆urkA::hph∆urhB::ble urhB^REC^*, *ptrA* | This study |
| *∆uprt∆urkA* | *∆uprt::ble∆urkA::hph* | This study |
| *∆uprt∆urhA* | *∆uprt::ble∆urhA::hph* | This study |
| *∆uprt∆urhB* | *∆uprt::ble∆urhB::hph* | This study |
| *∆uprt∆udpA* | *∆uprt::ble∆udpA::ptrA* | This study |
| *∆uprt∆udpB* | *∆uprt::ble∆udpB::hph* | This study |
| *∆uprt∆udpB udpB^REC^* | ∆*uprt::ble*∆*udpB::hph* *udpB^REC^*, *ptrA* | This study |

**References**

1. Fraczek MG, Bromley M, Buied A, Moore CB, Rajendran R, Rautemaa R, et al. The cdr1B efflux transporter is associated with non-cyp51a-mediated itraconazole resistance in *Aspergillus fumigatus*. J Antimicrob Chemother. 2013;68(7):1486-96. PMID: 23580559.

2. Birstonas L, Dallemulle A, Lopez-Berges MS, Jacobsen ID, Offterdinger M, Abt B, Straßburger M, Bauer I, Schmidt O, Sarg B, Lindner H, Haas H, Gsaller F. Multiplex genetic engineering exploiting pyrimidine salvage pathway-based endogenous counterselectable markers. mBio. 2020;11(2). PMID: 32265325.
