# Supplementary material for: The fungal expel of 5-fluorocytosine derived fluoropyrimidines mitigates its antifungal activity and generates a cytotoxic environment
Source: PLoS Pathog. 2022 Dec 27;18(12):e1011066. doi: 10.1371/journal.ppat.1011066 (PMC9829169; doi:10.1371/journal.ppat.1011066)
Supplement: S5 Table — (DOCX) [file ppat.1011066.s007.docx]

**S5 Table. Oligonucleotides used in this study.**

| **Primer set** | **Forward primer (5´ → 3´)** | **Reverse primer (5´ → 3´)** | **PCR product** |
| --- | --- | --- | --- |
| fcyA-1/2 | TTGAAACTCCGAGGAAGTCG | TAGTTCTGTTACCGAGCCGGTATGTGGATCCAGAGCGTCA | 5´ fcyA |
| fcyA-3/4 | GCTCTGAACGATATGCTCCCTTCGACAAAATGCCATTGAA | TACCTCCCCGAATACCATGA | 3´ fcyA |
| fcyA-N1/N2 | CGAGTCGCCTTAAAATGAGC | GTGGATCGGTATGCAGGATT | *fcyA* knock-in construct |
| uprt-1/2 | GGAAGGACAGGTACGCCATA | TAGTTCTGTTACCGAGCCGGCGGAGCACTCTGAAAATTGG | 5´ uprt |
| uprt-3/4 | GCTCTGAACGATATGCTCCCTCCCATCGTGTAGCGACATA | TACTACCTTCGCCCTCTGGA | 3´ uprt |
| uprt-N1/N2 | TTTGAGCGATTAAGGTGCAA | GCCCCACTACTTGTTTCCAG | *uprt* knock-in construct |
| urkA-1/2 | ATAGGTGGTAGGGCAGGAGG | TAGTTCTGTTACCGAGCCGGATTAGAATGCGGCGCAACAG | 5´ urkA |
| urkA-3/4 | GCTCTGAACGATATGCTCCCGGTCTATAGTGTCAGGCGGC | TACTACCTTCGCCCTCTGGA | 3´ urkA |
| urkA-N1/N2 | GCCAGAATGAATCGCAGTGC | TGCGATTCGTGACTTCTCCC | *urkA* deletion constructs |
| urhA-1/2 | TCACGCAATCTCTGCTCAAG | TAGTTCTGTTACCGAGCCGGGCCAGAATCCTCATGAAACC | 5´ urhA |
| urhA-3/4 | GCTCTGAACGATATGCTCCCGAACCTGGCGATCATAGACC | ACTGATCGGCTGACGTTTTT | 3´ urhA |
| urhA-N1/N2 | GGAGGCAACAATTCTTCCAA | TCACCAAGTGTGCTCGACTC | *urhA* deletion constructs |
| urhB-1/2 | AACTGAAGGAATCGCTGGAA | TAGTTCTGTTACCGAGCCGGTCGCATTACCCGTTTACTCC | 5´ urhB |
| urhB-3/4 | GCTCTGAACGATATGCTCCCAAGCATGCGCCTTTCATTAG | GAACGGGTCAATTGCGTATT | 3´ urhB |
| urhB-N1/N2 | CGGAGTAGCACTGGGAAGTC | GTGCTGATAGCGGAAGGAAG | *urhB* deletion constructs |
| udpA-1/2 | GGATATCGATCCGACTCTCG | TAGTTCTGTTACCGAGCCGGACGAGGCCAACAACAACAA | 5´ udpA |
| udpA-3/4 | GCTCTGAACGATATGCTCCCCTTCTCGTGGAAGTGGTACTG | TCCGAACTGAAAGCCTTTGT | 3´ udpA |
| udpA-N1/N2 | TTCCAAACCCTAATGCCAAG | TGCTGGTCTGACAATCGAAG | *udpA* deletion constructs |
| udpB-1/2 | GTGGTACGGCAGTGAGCAGT | TAGTTCTGTTACCGAGCCGGACAAATCGTGGGAACGAGAC | 5´ udpB |
| udpB-3/4 | GCTCTGAACGATATGCTCCCGCATCCCTTGCATCCATAGT | CAAGTTCAGCAAGGGGGTTA | 3´ udpB |
| udpB-N1/N2 | TTTATGGCCCGATTGCTTAG | GGGGCTCGGTCATAGTAGGT | *udpB* deletion constructs |
| urkAcompl-FW/RV | CCGGCTCGGTAACAGAACTATCCCACCGAGTGTATGATGA | GGGAGCATATCGTTCAGAGCCTTGTTCGTCGCGAAAATCT | Insert for pSK275 backbone (*urkA*) |
| urhBcompl-FW/RV | CCGGCTCGGTAACAGAACTAAGCGACCAGCTCTTTACCAA | GGGAGCATATCGTTCAGAGCCCCGAAGAAAAAGAGACAGC | Insert for pSK275 backbone (*urhB*) |
| udpBcompl-FW/RV | CCGGCTCGGTAACAGAACTACCCAGAAGACGGCCTATGAT | GGGAGCATATCGTTCAGAGCCGGTATAGTCCTCTGGGCAG | Insert for pSK275 backbone (*udpB*) |
| BBpSK275-FW/RV | GCTCTGAACGATATGCTCCCGCTTATCGATACCGTCGACCT | TAGTTCTGTTACCGAGCCGGAATGCCCCACCGTTACATAC | pSK275 backbone for genetic complementations |
| hph-FW/RV | CCGGCTCGGTAACAGAACTAACGGCGTAACCAAAAGTCAC | GGGAGCATATCGTTCAGAGCTCTTGACGACCGTTGATCTG | *hph* and *ble* cassettes |
| ptrA 5/3 | CCGGCTCGGTAACAGAACTAGCATCCCATTGGTAACGAAA | GGGAGCATATCGTTCAGAGCAATGCCCCACCGTTACATAC | *ptrA* cassette |
