# Supplementary material for: The fungal expel of 5-fluorocytosine derived fluoropyrimidines mitigates its antifungal activity and generates a cytotoxic environment
Source: PLoS Pathog. 2022 Dec 27;18(12):e1011066. doi: 10.1371/journal.ppat.1011066 (PMC9829169; doi:10.1371/journal.ppat.1011066)
Supplement: S6 Table — (DOCX) [file ppat.1011066.s008.docx]

**S6 Table. Plasmids used in this study.**

| **Plasmid** | **Used for** | **Reference** |
| --- | --- | --- |
| pAN7-1 | *hph* cassette amplification | (1) |
| pAN8-1 | *ble* cassette amplification | (2) |
| pSK275 | Amplification of pSK275-backbone and *ptrA* cassette | (3) |
| pFG66 | *urkA* reconstitution | This study |
| pFG67 | *urhB* reconstitution | This study |
| pESV38 | *udpB* reconstitution | This study |

**References**

1. Punt PJ, Oliver RP, Dingemanse MA, Pouwels PH, van den Hondel CA. Transformation of *Aspergillus* based on the hygromycin B resistance marker from *Escherichia coli*. Gene. 1987;56(1):117-24. PMID: 2824287.

2. Mattern IE PP, Van den Hondel CAMJJ. A vector for *Aspergillus* transformation conferring phleomycin resistance. Fungal Genet Rep. 1988;35(187):79-89.

3. Krappmann S, Jung N, Medic B, Busch S, Prade RA, Braus GH. The *Aspergillus nidulans* F-box protein GrrA links SCF activity to meiosis. Mol Microbiol. 2006;61(1):76-88. PMID: 16824096.
